# Physiological Maturity Determination of Wild Banana ( Musa acuminata subsp. malaccensis (Ridl.) N.W.Simmonds) for Seed Conservation

**DOI:** 10.12688/gatesopenres.16375.1

**Published:** 2026-01-12

**Authors:** Sahromi Sahromi, Aulia Hasan Widjaya, Dian Latifah, Kurniawati Purwaka Putri, Evayusvita Rustam, Muhammad Zanzibar, Yulianti Yulianti, Dwi Murti Puspitaningtyas, Lulut Dwi Sulistyaningsih, Irvan Fadli Wanda, Dewi ayu Lestari, Lia Hapsari, Enny Sudarmonowati, Apriliana Dyah Prawestri, Fajarudin Ahmad, Witjaksono Witjaksono

**Affiliations:** 1 National Research and Innovation Agency Republic of Indonesia, Bogor, West Java, Indonesia; 2 National Research and Innovation Agency Republic of Indonesia, Bogor, West Java, Indonesia; 3 National Research and Innovation Agency Republic of Indonesia, Bogor, West Java, Indonesia

**Keywords:** ethylene, germination, harvesting time, leachate conductivity, seed viability

## Abstract

The seed conservation and germination techniques for wild banana seeds are less understood, making this research crucial for developing high-quality seeds that can support sustainable banana breeding efforts. This study examined the reproductive biology and physiological maturity of *Musa acuminata* subsp. *malaccensis* to understand seed germination and seedling vigor in order to enhance banana breeding. This research focused on identifying the ideal harvest period to obtain seeds with optimal viability and how ethylene application affects seed quality. Wild banana accessions from the Cibinong Germplasm Collection were used in this study. Ethylene was used to accelerate fruit ripening. Observations were made on the vegetative and generative growth phases and fruit morphology. The physical and physiological qualities of wild banana seeds, M. *acuminata* subsp. *malaccensis* can be improved by harvesting between 71-90 days after receptive (DAR). Harvesting during this period increases seed viability and vigor compared to seeds harvested at 50-70 DAR, which showed the highest leachate conductivity value of 968.79 μS g ^−1^.

**Abbreviations**
%: Percent
μS g ^−1^
: microsiemen by gram
°C: Degree Celcius
ANOVA: analysis of variance
BBCH-Scale
: Biologische Bundesanstalt, Bundessortenamt dan CHemische Industrie-Scale
BBTV: Banana Bunchy Top Virus
BPS: Badan Pusat Statistik
BRIN: Badan Riset dan Inovasi Nasional
Cm: Centimeter
D: Day
DAR: Days After Receptive
DMRT: Duncan’s Multiple Range Test
Et al: And others
Fig.: Figure
Fl: Flowers
Fr: Fruits
g: gram
GC: germination capacity,
GP: germination percentage,
GR: germination rate
h: hour
ISTA: International Seed Testing Association
KST: Kawasan Sains dan Teknologi
LC: leachate conductivity
LIPI: Lembaga Ilmu Pengetahuan Indonesia
LPDP: Lembaga Pengelolaan Dana Pendidikan
mL L ^−1^
: Milliliter
mm: Millimetre
N: number
O: Ovuls
R ^2^
: the coefficient of determination in a regression model
RIIM: Riset Inovasi Untuk Indonesia Maju
RS: Reproductive success
S: Seeds
SAS: Statistical Analysis System
SE: standard of error
Subsp: Subspecies
T: Time
TSS: total soluble solids
TTA: total titratable acidity
w/v: weight-to-volume ratio

## Introduction

Currently, the commercial banana market is dominated by a limited number of cultivars, mainly Cavendish, plantains, and cooking bananas, resulting in reduced genetic diversity among the cultivated types. This lack of diversity increases vulnerability, as a single pest or disease can significantly affect banana production ( Ordonez et al., 2015; Ploetz, 2015; Varma & Bebber, 2019; Fones et al., 2020). This situation is exacerbated by the high sterility of cultivated bananas, which makes breeding efforts challenging ( Silva et al., 2001). In contrast, wild banana species possess fertile traits and have been successfully used as parents to produce promising offspring. These wild relatives are essential for developing improved diploid varieties in breeding programs ( Bakry et al., 2009). Consequently, the wild banana germplasm is a vital genetic resource for enhancing resistance to biotic and abiotic stresses and for acquiring other desirable traits ( Ramirez et al., 2011; Jones & Daniells, 2018).

Genetic erosion of wild banana germplasm occurs because of escalating environmental changes ( Hardaningsih et al., 2012), such as habitat destruction, fragmentation, and land degradation ( Riordan & Nabhan, 2019). This highlights the critical need for conservation efforts to preserve wild banana genetic resources, which are essential for the genetic improvement of bananas.

Wild banana seed conservation aims to preserve the genetic diversity of these plants in the long term, ensuring that they remain available for future use. Conserving banana seeds has become a global priority for food security ( Castaneda-Alvarez et al., 2016; Kallow et al., 2022). Indonesia is home to 12 species of wild banana out of 71 species worldwide, accounting for approximately 12.9% ( Poerba et al., 2016). Among these, the wild species *Musa acuminata* Colla (genome A) and *M. balbisiana* Colla (genome B) are particularly significant, as they are the wild relatives of cultivated bananas ( Simmonds, 1962).

The success of seed conservation is closely linked to a reliable seed procurement system that can consistently produce seeds with high physical, physiological, and genetic qualities. Access to high-quality seeds with significant genetic diversity is essential for successful seed conservation efforts ( Wambugu et al., 2023). Various technologies are available to ensure seed quality. Therefore, it is important to focus on seed technology for wild bananas, particularly in breeding efforts to develop resistance to *Fusarium* fungus and Banana Bunchy Top Virus (BBTV), which are currently major threats to banana crops worldwide ( Drenth & Kema, 2021). In addition to supporting the improvement of banana quality, this research is also important for conserving wild banana germplasms. Improving the quality of bananas will be part of supporting food security.

Seed technology involves processes that begin with seed production and extend through testing and storage, ensuring that the quality of the seeds is maintained. Seed quality is influenced by the timing of the harvest, specifically when the seeds reach physiological maturity. Seed ripening occurs from the completion of fertilization until harvest, and typically, seed maturity aligns with fruit maturity ( Vera Cruz et al., 2018). At this stage of maturity, seeds are considered physiologically mature and possess optimal food reserves to support sprout growth and achieve maximum germination capacity ( Sanoto et al., 2017).

The main challenge in wild banana development is the incomplete mastery of techniques for harvesting, storing, and germinating seeds ( Kallow et al., 2020, 2021). Harvesting issues such as determining the physiological maturity of seeds are poorly understood. Similarly, storage methods remain underdeveloped owing to the limited knowledge of seed characteristics. Germination also poses difficulties, such as asynchronous germination and dormancy symptoms, which are still not fully understood ( Chin, 1996; Nagano et al., 2009). The goal was to establish criteria for the physiological ripening phase of wild banana seeds, specifically *M. acuminata* subsp. *malaccensis* by observing the period of physiological maturity under natural conditions.

## Materials and methods

### Materials

**Figure 1.**
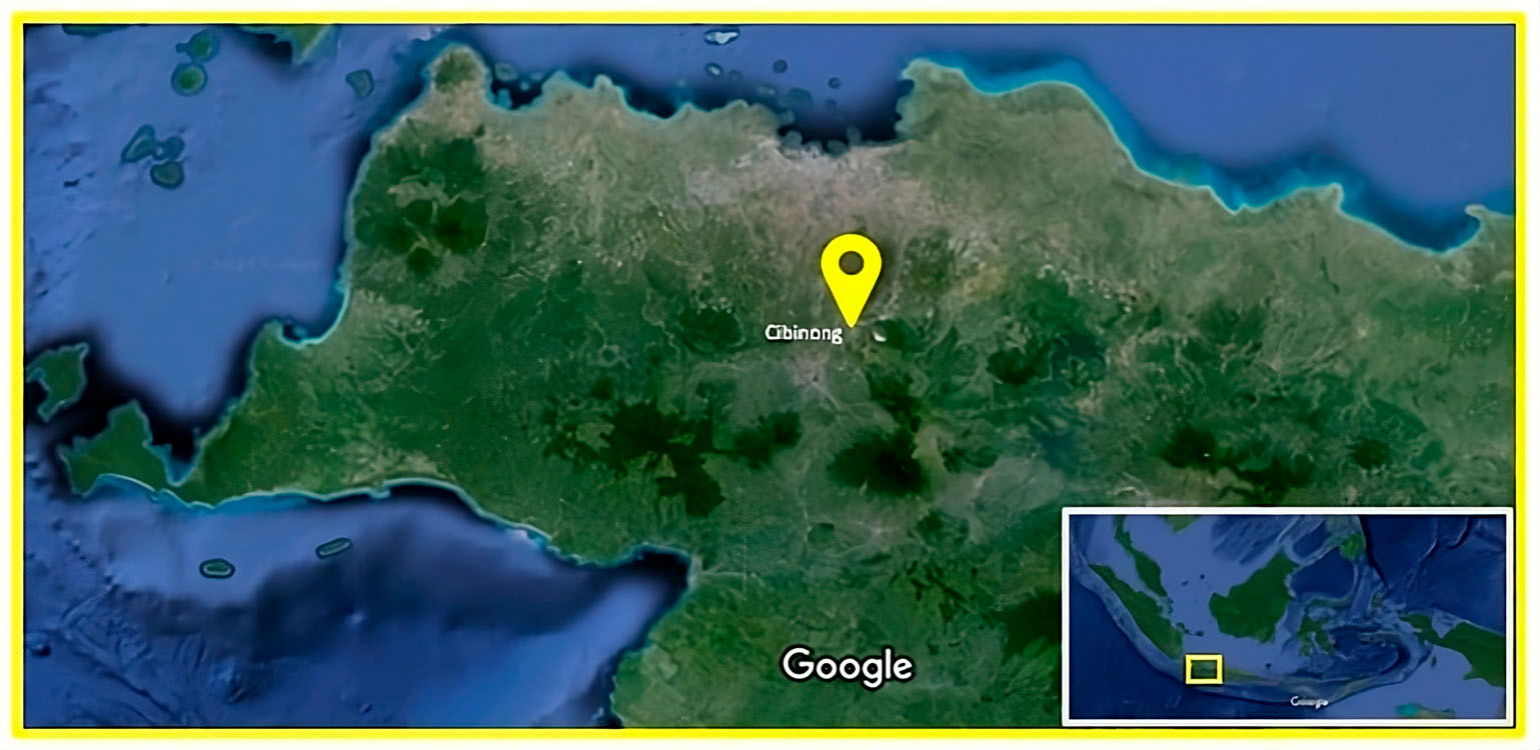
Map of research location at the wild banana germplasm collection garden in Cibinong, West Java, Indonesia.

The plant accession used was the wild banana plant accession *M. acuminata* subsp. *malaccensis*, from the wild banana germplasm garden at KST Soekarno Cibinong, West Java, Indonesia, which is located at the geographical coordinates of latitude -6.30429 and longitude 106.50412 ( Figure 1). The site is 168 m above sea level, with an average rainfall of 3153 mm/year and daily average temperature of 34 °C ( BPS, 2023). Twenty accessions were used in the present study ( Table 1). Two accessions were derived from open pollination of the LIPIX01 accession from KST Soekarno in Cibinong, West Java, Indonesia, whereas the remaining 18 accessions resulted from open pollination of the LIPI 01 accession collected in South Sumatra, Indonesia ( Poerba & Ahmad, 2010). The number of accessions was determined based on the availability of plants during the observation period. All plants were maintained under the same treatment conditions and consistent geo-climatic conditions.

**Table 1.** The source of *M. acuminata* subsp. *malaccensis* used in research at wild banana germplasm collection garden in Cibinong, West Java, Indonesia.

No	Accession	Accession source	Vegetative phase	Generative phase (flowering development)	Observation of fruit morphology
1	8A	Progeny from open pollinated *M. acuminata* subsp *. malaccensis* accession LIPI-010 BRIN collection in Cibinong			√
2	9A	Progeny from open pollinated *M. acuminata* subsp *. malaccensis* accession LIPI-010 BRIN collection in Cibinong			√
3	6B	Progeny from open pollinated *M. acuminata* subsp *. malaccensis* accession LIPI-010 BRIN collection in Cibinong			√
4	9B	Progeny from open pollinated *M. acuminata* subsp *. malaccensis* accession LIPI-010 BRIN collection in Cibinong			√
5	1C	Progeny from open pollinated *M. acuminata* subsp *. malaccensis* accession LIPI-010 BRIN collection in Cibinong	√		
6	6C	Progeny from open pollinated *M. acuminata* subsp *. malaccensis* accession LIPI-010 BRIN collection in Cibinong			√
7	13C	Progeny from open pollinated *M. acuminata* subsp *. malaccensis* accession LIPI-010 BRIN collection in Cibinong			√
8	1D	Progeny from open pollinated *M. acuminata* subsp *. malaccensis* accession LIPI-010 BRIN collection in Cibinong			√
9	6F	Progeny from open pollinated *M. acuminata* subsp *. malaccensis* accession LIPI-010 BRIN collection in Cibinong			√
10	9F	Progeny from open pollinated *M. acuminata* subsp *. malaccensis* accession LIPI-010 BRIN collection in Cibinong		√	
11	1G	Progeny from open pollinated *M. acuminata* subsp. *malaccensis* accession LIPI-X01 BRIN collection in Cibinong	√		
12	6G	Progeny from open pollinated *M. acuminata* subsp. *malaccensis* accession LIPI-010 BRIN collection in Cibinong		√	√
13	1H	Progeny from open pollinated *M. acuminata* subsp. *malaccensis* accession LIPI-010 collection BRIN di Cibinong	√	√	
14	8H	Progeny from open pollinated *M. acuminata* subsp. *malaccensis* accession LIPI-X01 BRIN collection in Cibinong		√	√
15	4I	Progeny from open pollinated *M. acuminata* subsp. *malaccensis* accession LIPI-010 BRIN collection in Cibinong	√		√
16	8I	Progeny from open pollinated *M. acuminata* subsp. *malaccensis* accession LIPI-010 BRIN collection in Cibinong		√	√
17	9I	Progeny from open pollinated *M. acuminata* subsp. *malaccensis* accession LIPI-010 BRIN collection in Cibinong			√
18	2J	Progeny from open pollinated *M. acuminata* subsp. *malaccensis* accession LIPI-010 BRIN collection in Cibinong			√
19	5J	Progeny from open pollinated *M. acuminata* subsp. *malaccensis* accession LIPI-010 BRIN collection in Cibinong			√
20	7J	Progeny from open pollinated *M. acuminata* subsp. *malaccensis* accession LIPI-010 BRIN collection in Cibinong			√

Notes: LIPI-010 accessions were from South Sumatra [13]; LIPI-X01 accessions were a landrace of wild bananas at KST Soekarno - Cibinong.

### The development of vegetative and generative phases

Vegetative growth began with the emergence of a vegetative bud, seen as a brownish-purple sheath on the soil surface, and continued until the appearance of a flag leaf, a short, stubby little leaf, heralding the emergence of the flower bud. Generative observations focused on tracking flowering development and fertilization success. Flowering development was monitored from the appearance of a flower bud until the fruit reached maturity, as indicated by the fruit skins turning yellow. The observed parameters included bunch length, number of opening bracts, rachis length, fruit length and diameter, flower length and diameter, and fruit apex length. The reproductive success rate was calculated using equation (1) from ( Owen et al., 1991). Generative phase observations were conducted thrice a week during the flowering stage and once a week during the fruit development stage.

$$\mathrm{RS}=\frac{\mathrm{Fr}}{\mathrm{Fl}}\;\times \;\frac{S}{O}$$ (1)

Where *RS* is the reproductive success rate, *Fr* is the number of fruits per hand, *Fl* is the number of flowers per hand, *S* is the number of seeds per fruit, and *O* is the number of ovules per flower.

Following harvest, wet seed extraction was performed to remove the pulp from the seeds. The observed parameters included fruit and seed length, diameter, and weight as well as the number of fruits in the hand and the number of seeds per fruit. Each treatment consisted of four repetitions with 25 observation units per repetition.

### Observation of fruit and seed morphology resulting from hormone application

In this study, ethylene was applied to stimulate fruit ripening via a spraying technique using a solution of 1 mL L ^−1^ ethylene directly onto the fruit while it was still attached to the tree. Ethylene treatment modified from ( Cahyono, 2009) was carried out at two different times starting from the receptive period of the female flower, namely 50-70 and 71-90 days after receptive (DAR). Subsequently, a transparent plastic lid with holes was placed over the bunch. Harvesting occurred five days post-spray, as indicated by the yellowing of the fruit skin. Following harvest, wet seed extraction was performed to remove the pulp from the seeds. The observed parameters included finger and seed length, diameter, and weight, as well as the number of fingers in the hand and number of seeds per finger. Each treatment consisted of four repetitions with 25 observation units per repetition.

### Assessment of seed physiological quality

The harvested seeds were used for physiological quality testing, including moisture content, germination capacity, germination percentage, germination rate, and leachate conductivity. The seed moisture content was determined using the direct method, with an oven set to a constant temperature of 103 ± 2 °C for 17 ± 1 h ( ISTA, 2018), and each sample consisted of 5 g of seeds. Germination capacity, germination percentage, and germination rate were measured using Equations (2-4) by ( Czabator, 1962; Sudrajat et al., 2022).

$$\mathrm{GC}=\frac{\text{number of seedling}}{\text{number of sown seeds}}\times 100\%$$ (2)

$$\mathrm{GP}=\frac{\text{number of normal seedling}}{\text{number of sown seeds}}\times 100\%$$ (3)

$$\mathrm{GR}=\frac{N1}{D1}+\frac{N2}{D2}+\ldots +\frac{\mathrm{Nn}}{\mathrm{Dn}}\;$$ (4)

where, *GC* is germination capacity, *GP* is germination percentage, *GR* is germination rate, *N* is the number of seeds that germinate in a particular unit of time ( *n* ^th^ day), *D* is the day of observation, and *T* is the amount of time (usually in days) between the start and end of the testing interval. Germination was observed when no additional germination occurred for four consecutive days ( Xu et al., 2016). The criterion for normal seedlings was the emergence of healthy leaves. Leachate conductivity was measured by soaking the seeds in ion-free water (Aquabidest) for 24 h at a 1:10 (w/v) ratio. After the seeds were removed, the conductivity of the remaining liquid was measured using a conductivity meter ( Zanzibar, 2016).

### Data analysis

Flower and fruit development observations were analyzed descriptively through digital camera documentation, whereas fruit and seed morphology and physiological quality were assessed using analysis of variance (ANOVA). If significant differences were found between treatments, Duncan’s Multiple Range Test (DMRT) was applied for further analysis at a 5% significance level. These analyses were performed using the SAS software (version 9.4; SAS, 2014). Physiological quality analysis used correlation tests to evaluate the relationship between moisture content, germination capacity, and leachate conductivity.

## Results and Discussions

### Development of the vegetative and generative phases

**Figure 2.**
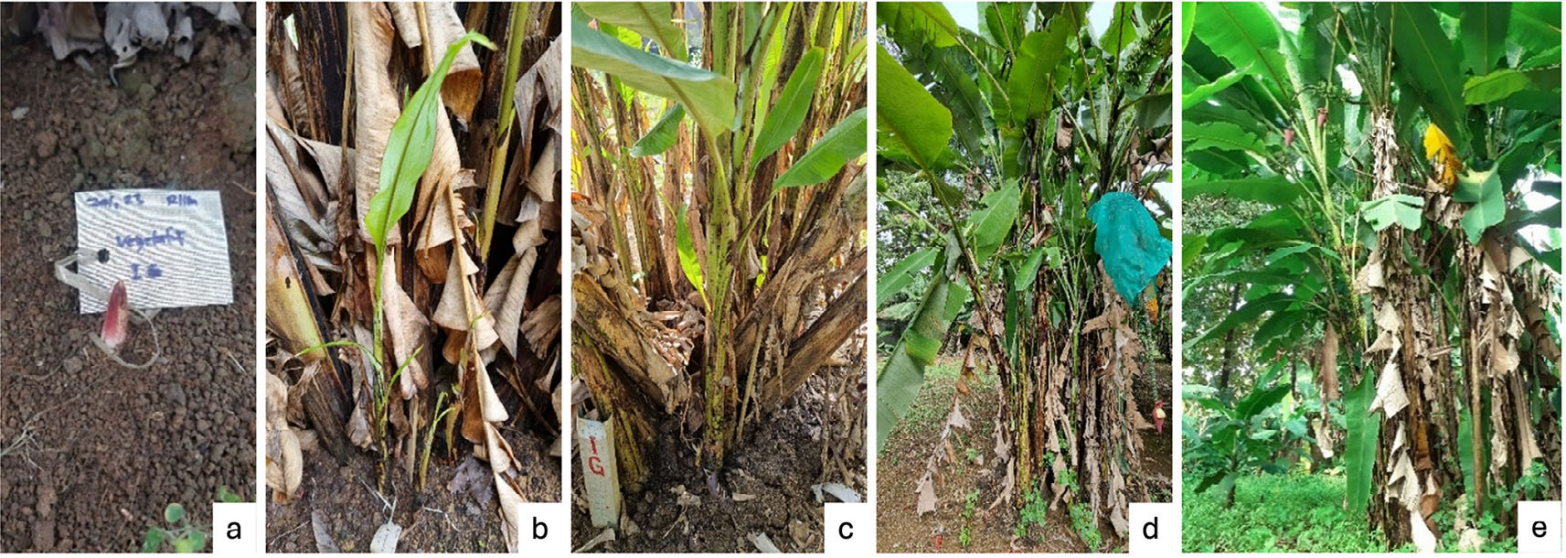
The development of vegetative shoots from a cluster, starting from the emergence of shoots on the soil surface (a); the leaf sheaths began to open, starting from the lowest part and continuing upwards (b); the stem diameter increased, the number of leaf sheaths grew, and the leaves reached their normal size by the mature stage (c); the stem diameter reached its maximum, and the leaf sheaths and leaves were fully developed (d); the plant reached the reproductive stage (e).

The vegetative phase of the wild banana, *M. acuminata* subsp. *malaccensis* began with the emergence of a vegetative shoot on the soil surface, occurring between days 1 and 6, followed by leaf development ( Figure 2a). Between days 7 and 60, the shoot grew into leaf blades, which unfolded from the lowest leaf sheath and continued upward ( Figure 2b). Further growth was observed through an increase in the pseudostem diameter, number of leaf blades, and leaf size, reaching maturity between days 61 and 210 ( Figure 2c). The generative phase was marked by the appearance of an inflorescence bud, which occurred between days 211 and 330 ( Figure 2d-e).

**Figure 3.**
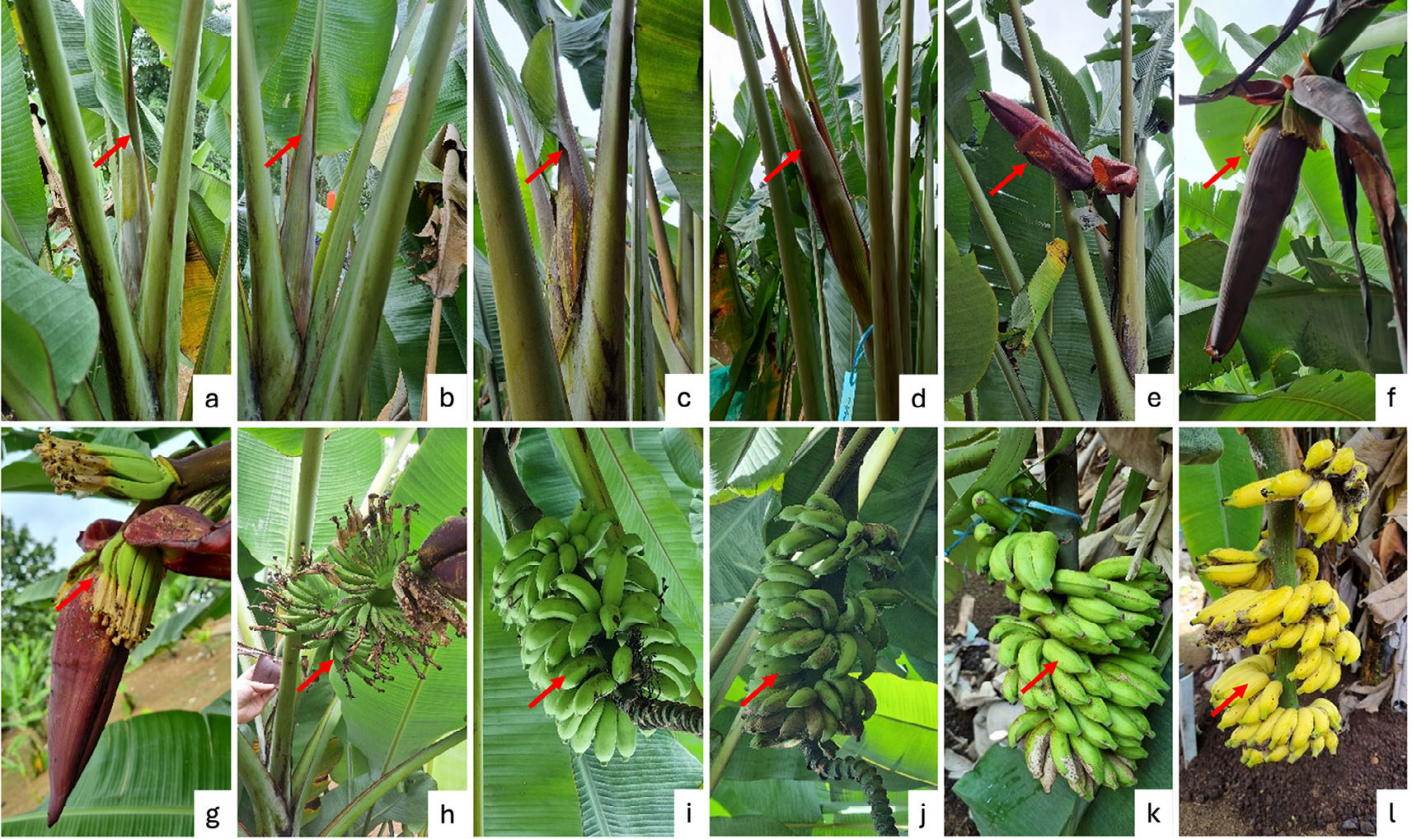
Generative development, from the emergence of the flower bud to fruit ripening. Various stages of growth and development of the flower bud (a-e); receptive flowers and male flower anthesis (f-g); fruit development until physiological maturity (h-l).

Figure 3 illustrates the developmental stages of the generative phase in the wild banana *M. acuminata* subsp. *malaccensis*, beginning with the emergence of an inflorescence bud, which was covered by a pair of greenish bracts. Between 3 and 5 days after receptivity (DAR), the bracts protecting the female flowers began to open and fall off, while the female flowers remained attached to the ovary, allowing pollination to occur. Following pollination, the female flowers attached to the fruit begin to wither and fall off. The fruits then continued to enlarge, with the filling process occurring between 9 DAR and 90 DAR. This is followed by seed maturation and fruit ripening, marked by the fruit peel changing color from green to yellow. Yellowing began around day 50 DAR and continued over a 10- to 20-day period until the entire peel turned yellow.

The development of wild banana *M. acuminata* subsp. *malaccensis* plants can be categorized into eight phases ( Meier, 2018; Widjaya et al., 2021). Based on the findings from this study, flower development leading to physiologically ripe fruit was divided into five phases (code 5-9), as shown in Table 2.

**Table 2.** Development phases of wild banana *M. acuminata* subsp. *malaccensis* adopted from BBCH-Scale.

Phase	Code	Description	Period of shoot emergence (days)	Image
**Vegetative phase**				
Sprouting/bud development	0	Vegetative shoot begins to emerge from the soil surface	1-6	2a
Leaf development	1	Leaf sheaths begin to open, starting from the lowest part and continuing upward until they reach normal size	1-7	2b
Shoot development	3	Stem diameter increases, and the number of leaf sheaths grows; the leaves reach normal size by the mature stage	61-210	2c
	4	Stem diameter reaches maximum, and leaf sheaths and leaves reach normal/mature size. Flower bud begins to emerge	211-330	2d-e and 3a,c
**Generative phase**				
Inflorescence emergence	5	Banana heart, first bract opens	1-2	3d-e
Flowering	6	- The bract protecting the female flower starts to open and detach	5-8	3f-g
		- Pollination occurs while the female flower is still attached to the ovary		
		- The female flower begins to wilt and detach from the ovary	3-5	
Development of fruit	7	- The bracts covering the male flowers fall off one by one	9-90	3h-i
		- The ovary (developing fruit) enlarges and becomes fully formed		
Maturity of fruit and seed	8	Fruit ripening is indicated by the change in banana skin colour from green to yellow	50-90	3j-k
Fruit senescence	9	All banana skin turns yellow (physiological maturity)	70-90	3l

The observations of the generative phase variables, including flowering to fruit formation and fruit morphology, are presented in Table 3. A description and brief qualitative assessment, such as categorizing the values as large, medium, or small, were necessary to provide a more meaningful context to the data.

**Table 3.** The reproductive variables of *M. acuminata* subsp. *malaccensis* in Cibinong, West Java, Indonesia.

No	Variables	Average	Category
1	Inflorescence length (cm)	130 ± 22.74	Medium in size
2	Number of opening bracts	130 ± 28.11	Many in number
3	Rachis length (cm)	4.9 ± 1.07	Medium in size
4	Number of hands per bunch	5.67 ± 0.87	Few in number
5	Number of fruits per hand	18 ± 3.28	Medium in number
6	Fruit length (cm)	8.6 ± 0.82	Medium in size
7	Fruit diameter (cm)	6.1 ± 0.4	Medium in size
8	Fruit apex length (cm)	0.9 ± 0.13	Small in size
9	Male bud length (cm)	13 ± 2.13	Long in size
10	Male bud circumference (cm)	19 ± 2.05	Big in size

The results of the observations on the seed characteristics from the 9 accessions observed were presented in Table 4. These should be adequately described, including the range, mean, and standard error.

**Table 4.** Seed characteristics of nine accessions of *M. acuminata* subsp. *malaccensis* and their average values.

Parameters	Accession number									Mean ± SE
	1D	1H	5J	6B	6G	7J	8A	8H	10H	
Seed thickness (mm)	2.28	2.67	2.46	3.05	2.41	2.59	2.44	2.89	2.86	2.63 ± 0.26
Seed diameter (mm)	6.3	5.64	6.62	5.47	5.43	5.43	6.24	6.41	4.88	5.83 ± 0.58
Seed weight (g)	0.014	0.037	0.028	0.034	0.022	0.029	0.027	0.035	0.024	0.03 ± 0.01
Embryonated seed (%)	64	74	80	88	64	64	66	98	96	77.11 ± 14.00

Reproductive success reflects the proportion of pollinated flowers that successfully matured into ripe fruits. Table 5 shows the average reproductive success of fruits treated with ethylene compared to those that were not. The data indicated that the naturally developed fruits achieved the highest percentage of seed formation, at 91.8%, producing an average of 118.80 ovules and 109.30 seeds per fruit.

**Table 5.** Reproductive success of *M. acuminata* subsp. *malaccensis* with ethylene treatment and natural development.

Treatments	Number of flowers per hand	Number of fingers per hand	Fruit set (%)	Number of ovules per finger	Number of seeds per finger	Seed set (%)	Reproductive success (%)
Natural	15.8	15.8	95.4	118.8	109.3	91.8	87.58
Ethylene	15.5	15.5	94.5	121.5	101.1	83.58	78.98

Flowering and fruiting observations were conducted from March to December 2023, during which the dry season occurred between July and October, with an average daily rainfall of 54.25 mm ( BPS, 2023). Climate was conducive to vegetative and generative growth, facilitating the comprehensive monitoring of all stages. Strong vegetative growth significantly aids in generative development ( Sulistiawati et al., 2017). Vegetative growth started with the emergence of shoots and leaf development, eventually reaching the maximum stem diameter and fully developed leaves within 210 days. The plants transitioned into generative or reproductive phases from day 211 to 310.

The banana inflorescence was arranged in a spiral pattern across the hands, with each hand enclosed by a protective bract. Female flowers develop first, followed by male flowers that remain enclosed by bracts called banana male buds ( Supriyadi & Suyanti, 2008). The bracts fell off after the female flowers opened, allowing pollination to begin. In this study, the bracts were opened 3-5 days after anthesis. According to Supriyadi and Suyanti (2008), banana flowers typically bloom approximately 20 days after the flower bud emerges, with bracts opening every 1-2 days over a period of 7-10 days. Following pollination, the fruit ovary expanded starting on the 9th day after anthesis, continuing until the 90 ^th^ day.

Banana fruits are arranged in “hands,” and multiple hands form a bunch. Each bunch typically contains 5–15 hands, each holding 6–22 fruits, depending on the variety ( Cahyono, 2002). The fruit is an elongated and curved berry. *M. acuminata* produces relatively short, rounded fruits, averaging 8.6 ± 0.82 cm in length (ranging from 7.43 to 9.69 cm) and 6.1 ± 0.40 cm in diameter (ranging from 5.56 to 6.63 cm). Wild bananas usually have seeds in their flesh and are not parthenocarpic ( Cahyono, 2002).

The ripening of bananas and their seeds is marked by a color change from green to yellow. Yellowing begins at approximately day 50 and is completed within 10-20 days, with full yellowing achieved by day 10. Ripening speed varies across banana subspecies and cultivars. For instance, Pisang Raja takes approximately 12-15 days to ripen, whereas faster-ripening varieties such as Cavendish ripen within 5-7 days ( Lustriane et al., 2018). Bananas are typically harvested 80-90 days after the last female flower blooms. For Pisang Barangan, the harvest period was between 68-88 days after flowering, based on changes in fruit weight, peel firmness, vitamin C content, total soluble solids (TSS), total titratable acidity (TTA), and TSS/TTA ratio. However, no significant changes were observed in flesh firmness or edible portions ( Widodo et al., 2019). The use of days after flowering to determine harvest time leads to variations in fruit ripeness.

A common approach for determining the harvest time for bananas is counting the number of days since flowering, referred to as the physiological method, which is fairly straightforward. Bananas are usually ready for harvest approximately 100 d after flowering ( Prabawati et al., 2008). In this study, wild bananas ripened 71-90 days after flowering, as indicated by the yellowing of the peel, suggesting that their harvest period was quicker than that of cultivated bananas.

### Fruit and seed morphology

Analysis of variance revealed that the interaction between the day after receptive and ethylene application had a significant impact on fruit morphology, including length, diameter, and weight. but not significant for apex length. Ethylene application significantly affected the fruit diameter. Ethylene treatment had a significant effect on seed length and diameter, but not on seed weight. The single factor of day after receptivity and the interaction between these two factors had no significant effect on seed morphology.

Naturally ripened fruits (without ethylene treatment) harvested at 71-90 DAR exhibited larger morphological traits, significantly differing from other treatments, with an average finger length of 9.41 ± 0.82 cm, a diameter of 21.30 ± 3.34 cm, a weight of 24.23 ± 7.16 g, and the shortest apex length of 0.67 ± 0.42 cm ( Table 6). In contrast, fruits treated with ethylene had the highest measurements at 50-70 DAR, showing average lengths, weight and apex length of 10.33 ± 1.33 cm; 23.71 ± 3.42 g, and 0.93 ± 0.24 cm, respectively but the biggest fruit diameter was found at 71-90 DAR, that is 18.00 ± 1.29 cm.

**Table 6.** The average of fruit and seed length, diameter and weight influenced by the bunch age.

Treatment	Harvest time	Seed length (cm)	Seed diameter (cm)	Seed weight (g)	Finger length (cm)	Finger weight (g)	Finger diameter (cm)	Apex length (cm)
Natural	50-70	0.43 ^a^ ± 0.06	0.30 ^a^ ± 0.03	0.03 ^a^ ± 0.01	8.46 ^bc^ ± 0.91	10.08 ^b^ ± 2.00	15.55 ^b^ ± 2.68	1.13 ^a^ ± 0.21
	71-90	0.48 ^a^± 0.07	0.29 ^a^ ± 0.02	0.03 ^a^ ± 0.003	9.41 ^ab^ ± 0.82	24.23 ^a^ ± 7.16	21.30 ^a^ ± 3.34	0.67 ^a^ ± 0.42
Ethylene	50-70	0.53 ^a^ ± 0.11	0.26 ^a^ ± 0.07	0.06 ^a^ ± 0.01	10.33 ^a^ ± 1.33	23.71 ^a^ ± 3.42	6.76 ^c^ ± 0.28	0.93 ^a^ ± 0.24
	71-90	0.53 ^a^± 0.13	0.23 ^a^ ± 0.06	0.04 ^a^ ± 0.02	7.52c ± 0.62	12.46 ^b^ ± 2.36	18.00 ^a^ ± 1.29	0.70 ^a^ ± 0.11
F Value		0.43 ns	1.21 ns	0.06 ns	38.76 *	88.91 *	14.99 *	1.84 ns

Physical, physiological, and biochemical changes characterize the seed maturation process. Physiological indicators of seed maturity can generally be observed through the physical appearance of the fruit, such as changes in peel color ( Santos et al., 2020), the formation of a “black layer” in corn ( Nleya et al., 2019), the emergence of a distinct aroma and fruit cracking in forest plants ( Kaiser et al., 2016), and a decrease in seed moisture content in wheat ( Calderini et al., 2000). Other visual signs of physiological seed maturity can also be indicated by the sizes of the fruit and seeds. Typically, seeds reach their maximum size when physiologically mature ( Suita & Megawati, 2009; Suharsi et al., 2015).

**Figure 4.**
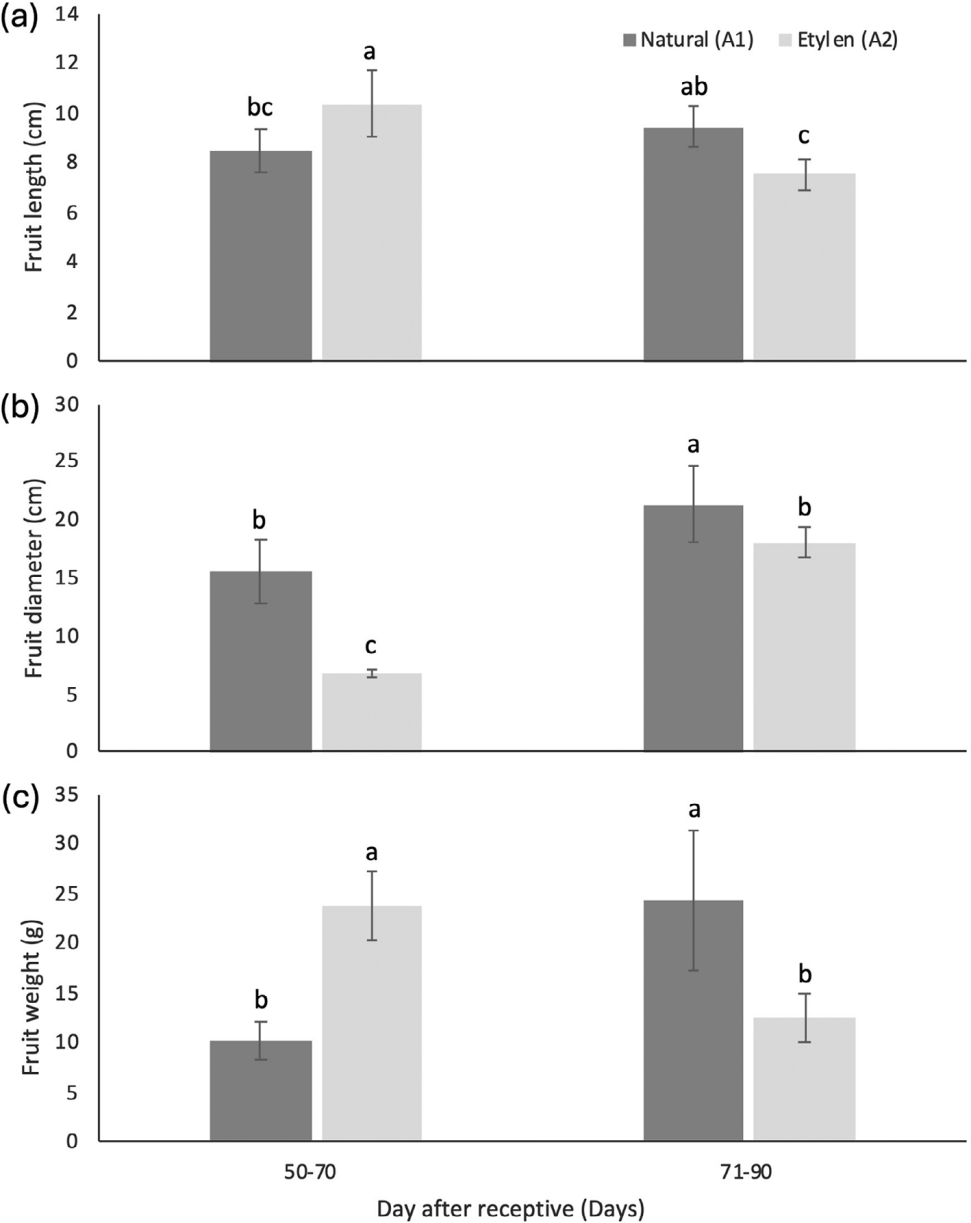
The average length of fruit (a), diameter of fruit (b), and weight of fruit of *M. acuminata* subsp. *malaccensis* seeds are treated on different days after receptivity and combined with different ethylene treatments. The same letters within the same chart are not significantly different at a 95% confidence interval, a<b<c.

**Figure 5.**
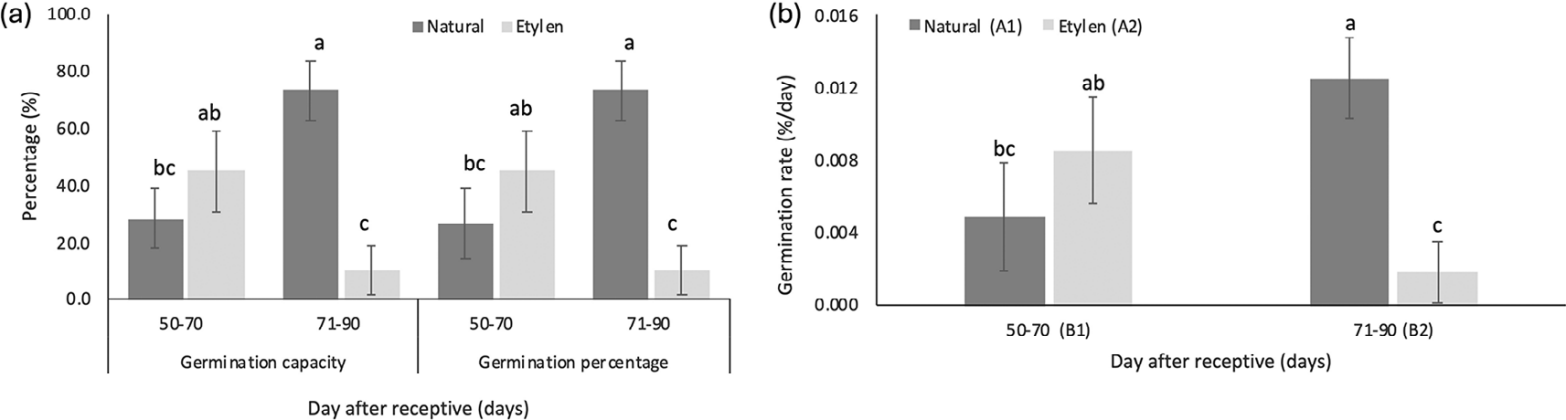
The average of germination capacity and germination percentage (a), and germination rate (b) of *M. acuminata* subsp. *malaccensis* seeds are treated on different days after receptivity and combined with different ethylene treatments. The same letters within the same chart are not significantly different at a 95% confidence interval, a<b<c.

The results of the study indicated that the largest fruit size—in terms of length, diameter, and weight—was observed in naturally ripened fruits harvested between 71-90 days after flowering, with measurements of 9.41 ± 0.82 cm in length, 21.30 ± 3.34 cm in diameter, and 24.23 ± 7.16 g in weight ( Figure 4). In contrast, under ethylene treatment, the greatest fruit size (specifically in length and weight) occurred at an earlier harvest age of 50-70 days, measuring 10.33 ± 1.33 cm in length and 23.71 ± 3.42 g in weight. When comparing seed size between naturally ripened fruits and those subjected to ethylene treatment across different harvest times, no statistically significant differences were observed for any of the measured parameters ( Table 6). However, when considering germination capacity (GC) and germination percentage (GP), fruits harvested between 71 and 90 days under natural conditions—with the largest physical dimensions—exhibited the highest values for both GC and GP, each reaching 73.33 ± 10.41% ( Figure 5).

One factor influencing variations in seed weight is endosperm content. The endosperm plays a crucial role in germination success. It affects the ability of the seed to absorb water (imbibition) and provides potential chemical energy for germination ( Farooq et al., 2023). During the early stages of germination, the seed absorbs water through imbibition, which softens the seed coat, hydrates the protoplasm, and activates enzymes, particularly those involved in converting fat into energy via respiration ( Pompelli et al., 2023).

Seeds contain carbohydrates, proteins, fats, and minerals, which are crucial energy sources and raw materials for embryos during germination. Larger and heavier seeds are thought to store more food reserves than smaller seeds. In sorghum ( *Sorghum vulgare*) seeds, a larger embryo is positively associated with higher protein content; the bigger and heavier the seeds, the greater their protein content ( Sutopo, 2002).

### Seed physiological quality

The ethylene spraying treatment interacted significantly with the fruit harvest age treatment across all observation variables. While ethylene treatment notably influenced all parameters, harvesting time significantly affected leachate conductivity and moisture content, but did not influence GC, GP, and GR ( Table 7).

**Table 7.** F value and its significance level resulted from ethylene spraying on the fruit and harvesting time on the seed physiological qualities of *M. acuminata* subsp. *malaccensis.*

Source of variation	F value and its level of significance				
	Leachate conductivity (LC)	Moisture content (MC)	Germination capacity (GC)	Germination percentage (GP)	Germination rate (GR)
Ethylene spraying (A)	109.49*	606.31*	12.97*	10.70*	7.06*
Harvesting time (B)	118.73*	711.93*	0.60 ns	0.72 ns	2.99 ns
Interaction (A×B)	41.24*	884.69*	38.13**	35.23**	15.73*

**Figure 6.**
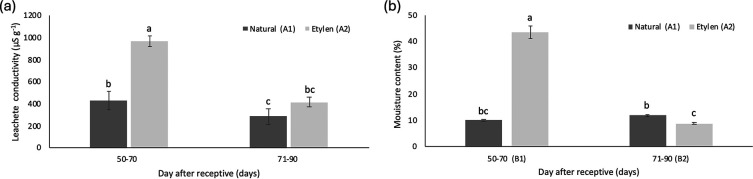
The average leachate conductivity (a) and moisture content (b) of *M. acuminata* subsp. *malaccensis* seeds are treated on different days after receptivity and combined with different ethylene treatments. The same letters within the same chart are not significantly different at a 95% confidence interval, a<b<c.

The leachate conductivity values of *M. acuminata* subsp. *malaccensis* seeds declined as fruit harvesting time increased, with a more notable reduction in seeds from ethylene-treated fruits. These conductivity values ranged from 968.79 μS g ^−1^ to 285.59 μS g ^−1^. Seeds from fruits harvested between 50-70 DAR, treated with ethylene, exhibited the highest average electrical conductivity at 968.79 ± 49.93μS g ^−1^ ( Figure 6a).

Overall, the moisture content of seeds ranged from 8.71% to 43.59%. For seeds from fruits treated with ethylene, moisture content generally decreased with increasing harvesting time, whereas moisture content in seeds without ethylene treatment tended to increase. The lowest seed moisture content, recorded at 8.71%, came from harvested fruits (71-90 DAR) with ethylene treatment ( Figure 6b).

Germination capacity and germination percentage of natural treatments showed higher value compared to ethylene treatments on harvesting time 71-90 DAR ( Figure 5a). The germination rate of seeds significantly increased from 0.126 %/day to 0.518 %/day ( Figure 5b) as the harvesting time progressed from 50-70 to 71-90 DAR in fruits not treated with ethylene. In contrast, seeds from ethylene-treated fruit tended to have lower germination rates ( Figure 5b).

**Figure 7.**
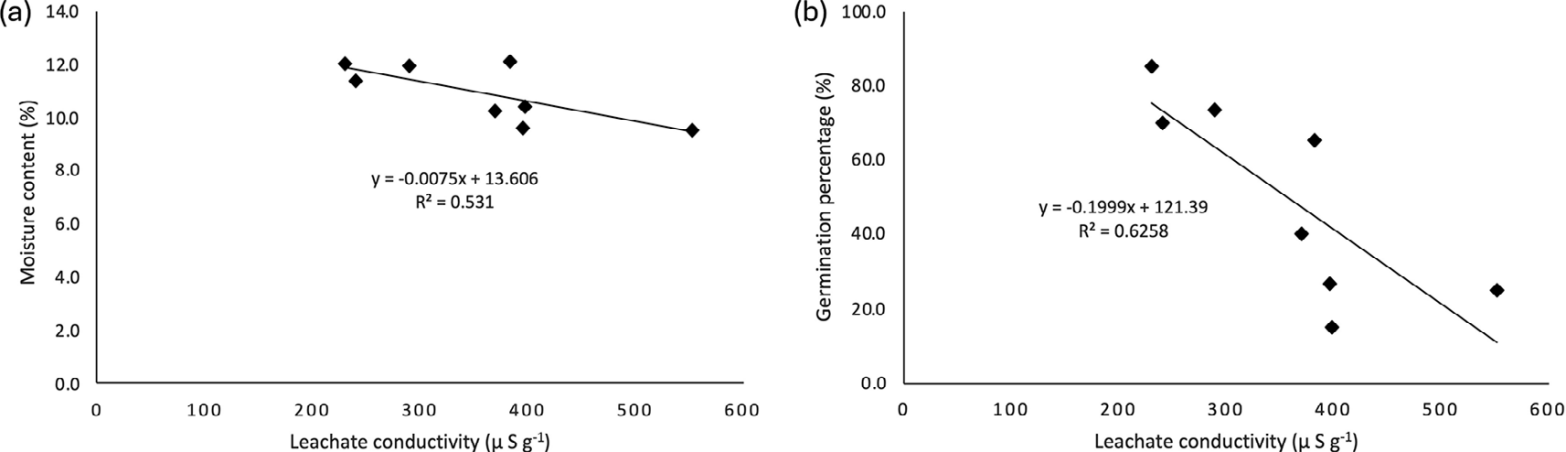
Correlation between leachate conductivity (LC), seed moisture content (MC) and germination percentage (GP).

As shown in Figure 7, seed moisture content (MC) exhibits negatively correlated with both electrical conductivity (LC) and germination percentage (GP). An increase in LC is associated with a decline in GP, whereas a decrease in MC leads to a corresponding increase in LC. Notably, LC appeared to be more sensitive to variations in GP than to changes in MC, suggesting a stronger predictive value of LC for seed vigor compared to moisture content alone. Wild banana seeds exhibited a negative correlation between MC and LC, with a coefficient of 0.531. This indicates that leachate conductivity could serve as a parameter for estimating the moisture content of wild banana seeds. The correlation between GP and LC was also negative (0.626) ( Figure 7).

The optimal time to harvest seeds of optimal quality is when the fruit reaches physiological maturity, with seed moisture content serving as an indicator of seed maturity ( Sripathy & Groot, 2023). In this study, the moisture content of naturally ripened *M. acuminata* seeds (without ethylene treatment) increased as the harvest time progressed. The highest seed moisture content, 11.88%) was recorded at 71-90 DAR without ethylene treatment. Changes in seed moisture during development are key factors influencing seed viability and storage characteristics ( Ghive et al., 2007). The moisture content stabilized between 50-70 and 71-90 DAR.

Physiological maturity can be indicated by seed viability and vigor, which are evaluated by germination capacity and germination rate. In this study, seeds harvested at 71-90 DAR likely achieved physiological maturity, with the highest germination percentage of 73.33% compared to other treatments. Additionally, seeds at 71-90 DAR not treated with ethylene demonstrated the best results regarding germination capacity and germination rate. The seeds probably possess fully developed food reserves at this maturity stage to facilitate seedling growth. Trimanto et al. (2022) reported that over 50% of seeds from mature *M. acuminata* subsp. *flava* fruits germinated at 61.33% ± 2.32% when tested on rice straw paper, whereas seeds from less mature fruits had a lower germination rate of 37.33% ± 1.01%.

Seed viability can indicate physiological maturity, which is evaluated by the germination capacity and germination percentage. In this study, seeds harvested at 71-90 DAR likely achieved physiological maturity, with the highest germination percentage of 73.33%. Seeds that germinate before physiological maturity typically exhibit a low viability. Harvesting fruit prematurely before reaching physiological maturity produces underdeveloped and not fully filled seeds, resulting in lower quality ( Suharsi et al., 2015). In this study, the low viability of seeds harvested at 50-70 DAR is likely attributable to immature embryo development.

Leachate conductivity serves as a measure of seed viability based on the idea that increased cell membrane leakage, reflected by higher electrical conductivity in the seed soaking solution, indicates lower seed viability due to membrane damage ( Noviana et al., 2016; Fatonah et al., 2017). High conductivity values suggest compromised membrane structure ( Nugraha & Wahyuni, 1998; Mavi et al., 2014), which can hinder germination ( Mavi et al., 2014). In this study, seeds harvested 71-90 DAR exhibited a conductivity of 285.59 μS g ^−1^ and a germination capacity of 73.33%. These results indicated that higher leachate conductivity is linked to reduced germination capacity, a trend also observed in *Kielmeyera coriacea* Mart ( Ramos et al., 2012). These findings imply that seeds from ethylene-treated fruits undergo delayed metabolic processes. Elevated leachate conductivity values corresponded to decreased seed viability, as indicated by lower germination capacity, establishing a negative correlation between leachate conductivity and germination capacity. Additionally, the average conductivity of seeds from ethylene-treated fruits was higher than that from naturally ripened fruits (691.83 μS g ^−1^). Correlation analysis also revealed a significant but negative relationship between leachate conductivity and germination capacity (R ^2^ = 0.6258) ( Figure 6).

## Conclusions

Developing the vegetative and generative phases of wild bananas can help to determine the optimal harvest time, indicating that the fruit has reached physiological maturity. The physical and physiological qualities of seeds from the wild banana *M. acuminata* subsp. *malaccensis* can be enhanced by harvesting between 71-90 days after reception. Harvesting during this period increases seed viability compared to seeds harvested at 50-70 days after receptive, which showed the lowest leachate conductivity value of 285.59 μS g ^−1^ and a germination capacity of 73.33%. Ethylene treatment did not have a positive effect on the physiological quality of *M. acuminata* subsp. *malaccensis* seeds across all harvest periods.

## Data Availability

Repositori Ilmiah Nasional (RIN): Data for: Physiological Maturity Determination of Wild Banana ( *Musa acuminata* subsp. *malaccensis* (Ridl.) N.W. Simmonds) for Seed Conservation. https://hdl.handle.net/20.500.12690/RIN/GFP47N ( Sahromi *et al*., 2025). This project contains the following underlying data:
- Figure 4 - Figure 5 - Figure 6 - Table 6 - Table 7 Data are available under the terms of CC BY-NC-SA 4.0.
